# Enhancing the Management of Opioid Use Disorder in the Emergency Department: A Holistic Curricular Intervention

**DOI:** 10.7759/cureus.76855

**Published:** 2025-01-03

**Authors:** Erin F Shufflebarger, Sherell Hicks, Stacy Marshall, Lauren A Walter

**Affiliations:** 1 Emergency Medicine, University of Alabama at Birmingham School of Medicine, Birmingham, USA

**Keywords:** curriculum development and evaluation, emergency medicine training, opioid use disorders, social emergency medicine, stigma and awareness

## Abstract

Introduction

Amid the opioid crisis, emergency medicine (EM) physicians increasingly tackle opioid use disorder (OUD) in the emergency department. Although the "X waiver" is no more, OUD-specific education requirements persist for EM clinicians and trainees. Traditional EM education emphasizes pathophysiology but overlooks the role of stigma in OUD as well as the importance of a peer recovery support specialist (peer) and community referral partners. We aimed to create and deliver a holistic EM OUD curriculum to boost practical OUD-specific knowledge and address OUD perceptions.

Methods

A task force of EM clinician-educators with expertise in OUD developed a four-hour educational curriculum to implement during a single EM residency didactic day. The curriculum highlighted a panel of peers, a series of lectures detailing specific aspects of OUD management, and an OUD tabletop simulation that focused on stigma. Pre- and post-participant surveys were obtained. Surveys assessed participant content knowledge via self-assessment as well as attitudes regarding persons with OUD via the Medical Condition Regard Scale (MCRS). A descriptive analysis was performed.

Results

Participants included 21 EM residents at various levels of training and faculty members, with 23 participants completing the pre-survey and 21 completing the post-survey. Post-curriculum delivery, all self-assessed knowledge categories demonstrated improvement; as examples, post-intervention, 100% of respondents reported understanding of medications for OUD (buprenorphine) indications and comfort with prescribing, compared to 52.7% and 56.5% prior to the intervention, respectively. Pre-survey MCRS assessments were largely positive; however, post-curriculum MCRS responses trended toward additional improvements in reported participant attitudes toward persons with OUD. A majority (95.2%) of respondents felt the curriculum improved topic understanding, and 90.5% reported improved confidence in treating patients with OUD. The peer panel and lectures were rated the most meaningful, followed by the simulation.

Conclusion

This OUD curricular intervention demonstrates the feasibility and perceived participant value of a holistic OUD curriculum. This curriculum improved participant OUD-specific knowledge and attitudes toward persons with OUD.

## Introduction

The opioid epidemic is a critical public health crisis in the United States, with overdose deaths surpassing 80,000 annually [1]. The impact of the opioid epidemic is seen daily in emergency departments (EDs) across the country, with patients frequently presenting for opioid-related conditions including overdose, withdrawal [2], and infections such as injection-site abscesses [2], endocarditis, and bacteremia [3]. In this unique position on the frontlines of the opioid crisis, emergency medicine (EM) physicians are encouraged to integrate non-opioid pain management strategies [4] while also engaging in comprehensive OUD management. This includes implementing harm reduction strategies, prescribing medications for opioid use disorder (MOUD) such as buprenorphine, and facilitating linkage to addiction management services [5]. The elimination of the “X-waiver” has removed a barrier to prescribing MOUD, but education on OUD management remains essential to ensure providers have the practical knowledge and confidence to manage OUD effectively [6].

Traditional EM training focuses on the pathophysiology and management of emergent and urgent complications of OUD. Non-medical factors that influence care, such as stigma, are often underemphasized [7]. Individuals with OUD often experience various forms of stigma, including social stigma, self-stigma, and structural stigma [8]. These stigmas can negatively affect their health and well-being, creating barriers to initiating treatment and often persisting even during recovery [8]. Addressing stigma in clinical education is critical, as negative perceptions may influence provider decision-making and the willingness to engage patients in treatment. Storytelling and the patient narrative are effective mechanisms to explore stigma and its effects [8]. In addition to medical management, it’s essential for EM providers to understand the role of peer recovery support specialists (peers) and community referral partners, as referral to and engagement in treatment is necessary to support long-term recovery. Previous literature suggests that learning opportunities and discussions with peers serve as a mechanism to enhance and sustain empathy among EM residents [9].

We aimed to create and deliver an updated holistic EM OUD curriculum to enhance practical OUD-specific knowledge, support the integration of community partners for comprehensive OUD management, and reduce stigma.

This work was presented as a poster abstract presentation at the Society for Academic Emergency Medicine (SAEM) Annual Meeting on May 16, 2024, in Phoenix, United States.

## Materials and methods

Study design and protocol

After the need for this curriculum was demonstrated [9,10], we established goals, objectives, and educational strategies to meet these objectives. The curriculum was then implemented and subsequently evaluated by the learners. This process aligned with Kern’s framework for curricular development [11].

A task force comprised of EM clinician-educators, including an EM toxicologist who specializes in substance use disorders, a social emergency medicine (SEM) fellowship director and SEM-trained faculty member, and an EM assistant program director, was assembled at the University of Alabama at Birmingham (UAB). The pilot “OUD curriculum day” was designed as a single didactic and experiential learning block. It included four continuous hours of resident education time plus 30 minutes to complete pre/post surveys, for a total of 4.5 hours. This study was reviewed and subsequently determined to be exempt by the UAB Institutional Review Board.

The final curriculum, detailed in Table 1, included three components: a peer panel, three subtopic lectures, and a tabletop simulation and debrief. The material for the subtopic lectures was chosen considering the patient population frequently encountered by the resident learners and, when replicated, can be adjusted to meet the needs of the learners and their surrounding community. The curriculum was delivered by members of the curriculum development task force along with invited OBGYN faculty and simulation faculty in December 2023.

**Table 1:** Components of holistic OUD curriculum day OUD: opioid use disorder, EM: emergency medicine, OBGYN: obstetrics and gynecology

Component	Description	Time allotted	To replicate
1. Peer panel	A panel of peer recovery support specialists living in active recovery shared insights from their personal and professional experiences. A question-and-answer format was used to explore topics including the role of a peer recovery support specialist, the experiences of individuals with OUD within the healthcare system (including barriers to access, challenges faced, and positive support received), and how EM physicians can more effectively serve and support patients with OUD.	60 minutes	Connect with local organizations or hospitals that provide addiction recovery services to identify peer support specialists who may be willing to share their experiences. Specific questions asked to the panel provided in Appendix A.
2. Subtopic lectures	PowerPoint slide presentations	90 minutes (30 minutes each)	Tailor topics to local community need.
	1. “5 Myths about opioids and OUD”		
	2. “OUD and Pregnancy” by OBGYN faculty		
	3. “Opioid Potpourri” by toxicology faculty		
3. Opioid simulation	Led by SimUAB, the opioid simulation is a tabletop simulation experience designed to help participants understand:	90 minutes	See [12] for additional sim information including facilitator training
	1. “OUD is a chronic, relapsing disease for which there is treatment and recovery”		
	2. “The role of stigma in the opioid epidemic”		
	3. “The impact of personal resiliency on opioid use”		

Study setting and population

The UAB Emergency Medicine Residency Program, located in Birmingham, Alabama, is a three-year residency program accredited by the Accreditation Council for Graduate Medical Education (ACGME), with 35 residents as of December 2023. Residents have protected time to participate in a structured weekly half-day didactic session, with excusal allowed under certain circumstances in accordance with ACGME guidelines [13]. The residents receive intermittent didactic education on OUD as part of the routine substance use disorder component of the curriculum, but they have not yet participated in dedicated, comprehensive OUD/MOUD training.

Key outcome measures

We developed two participant surveys, adapting previously used surveys with the authors’ permission [9], and distributed paper copies to participating UAB EM residents and faculty to evaluate the effect and impact of the curriculum as well as generate general feedback. All EM residents and faculty who attended the conference day were invited to participate voluntarily. Survey responses remained anonymous, with pre- and post-intervention surveys linked using a unique identifier. Data from participants who did not attend the entire conference day or failed to complete both pre- and post-surveys were excluded from the analysis. The surveys included general demographic information (e.g., age, gender, race) and subjective data measured on a Likert scale assessing participants’ self-perceived attitude and comfort level regarding recognizing and managing OUD in the ED setting. Additionally, both pre- and post-surveys also incorporated the Medical Condition Regard Scale (MCRS) to evaluate participants’ attitudes toward patients with OUD. The MCRS, previously validated in a similar population, measures “the degree to which respondents find patients with a given medical condition enjoyable, treatable, and worthy of medical resources” [14]. The surveys focused on participant reactions to and satisfaction with the curriculum as well as measuring learner attitude change as a result of the curriculum [15].

Data analysis

Paper surveys were distributed and collected in real time immediately before and after the conference day. This method was used to increase the response rate from participants and to ensure timely and quality feedback. Descriptive statistics, including frequencies and percentages, were calculated for categorical data. A paired sample t-test was conducted to determine whether there was a difference between matched pre- and post-survey responses from participants. A p-value of <0.05 is considered statistically significant. All statistical analyses were performed using JMP Pro 16 (JMP, Version 16, SAS Institute Inc., Cary, United States, 1989-2024). Paired MCRS responses were also compared to evaluate for any changes in participant regard after the curriculum.

## Results

A total of 23 participants completed the pre-conference survey, and 21 participants completed the immediate post-conference survey, with 18 surveys successfully matched using unique identifiers. Demographic data is shown in Table 2. Participants included eight PGY-1 residents, four PGY-2 residents, nine PGY-3 residents, and three EM attending physicians. The mean age of survey participants was 31.3, and a majority (73.1%) were male.

**Table 2:** Participant demographics PGY: postgraduate year

Demographic	n (%)
Gender	
Male	19 (73.1)
Female	7 (26.9)
Race	
White	24 (92.3)
Asian	1 (3.8)
Other	1 (3.8)
Age (years)	
Mean ± SD	31.3 ± 5.3
Median, range	29, 27-50
Training level	
PGY-1	8 (33.3)
PGY-2	4 (16.7)
PGY-3	9 (37.5)
Attending	3 (12.5)

Nearly half of the participants (45.5%) reported having prescribed MOUD to more than 10 patients in the ED, while two participants had never prescribed MOUD before. Participants identified many encountered or anticipated barriers to MOUD initiation in the ED, including provider time constraints, costs associated with MOUD, the challenge of recurrent ED visits for re-prescribing, and unfamiliarity with available resources for continued treatment.

As demonstrated in Table 3, participants generally agreed that the ED is an appropriate venue to engage patients with OUD in MOUD and felt comfortable, even before the curriculum, identifying OUD in the ED and recognizing signs and symptoms of opioid withdrawal. Participants reported significant improvement in their knowledge about buprenorphine’s indications, contraindications, and side effects. There was a significant improvement in participant comfort with prescribing MOUD in the ED, with 100% of respondents feeling comfortable with prescribing post-curriculum, compared to 52.7% pre-curriculum.

**Table 3:** Survey results (n (%)) *paired samples, p<0.05 ED: emergency department; OUD: opioid use disorder; MOUD: medications for opioid use disorder

Survey question	Pre-survey response (n=23)	Post-survey response (n=21)
The ED is an appropriate venue to engage patients with OUD with MOUD.		
Strongly agree/Agree	22 (95.7)	21 (100.0)
Strongly disagree/Disagree	1 (4.4)	0 (0.0)
I feel comfortable identifying and diagnosing OUD in the ED.		
Strongly agree/Agree	20 (87.0)	21 (100.0)
Strongly disagree/Disagree	3 (13.0)	0 (0.0)
I feel comfortable recognizing opioid withdrawal signs and symptoms.		
Strongly agree/Agree	22 (95.7)	21 (100.0)
Strongly disagree/Disagree	1 (4.4)	0 (0.0)
I understand buprenorphine (Suboxone) indications and contraindications as well as its clinical effects and side effects.*		
Strongly agree/Agree	12 (52.2)	21 (100.0)
Strongly disagree/Disagree	11 (47.8)	0 (0.0)
I feel very comfortable prescribing/providing buprenorphine (Suboxone) in the ED for opioid withdrawal.*		
Strongly agree/Agree	13 (56.5)	21 (100.0)
Strongly disagree/Disagree	10 (43.5)	0 (0.0)
I feel very comfortable providing an OUD-diagnosed patient with buprenorphine (Suboxone) at time of ED discharge.*		
Strongly agree/Agree	11 (47.8)	21 (100.0)
Strongly disagree/Disagree	12 (52.2)	0 (0.0)
I feel very comfortable instructing my patients on how to take buprenorphine (Suboxone) at home including initiation of home induction.*		
Strongly agree/Agree	9 (39.1)	21 (100.0)
Strongly disagree/Disagree	14 (60.9)	0 (0.0)
I am aware and familiar with local community resources to refer ED OUD patients to for a definitive substance use treatment including MOUD.*		
Strongly agree/Agree	9 (39.1)	20 (95.2)
Strongly disagree/Disagree	14 (60.9)	1 (4.8)

Table 4 shows the MCRS survey results, which measured participant attitudes toward patients with OUD before and after the curriculum. Pre-survey results were largely positive, indicating generally favorable attitudes toward patients with OUD. Post-curriculum MCRS responses showed an additional positive shift, suggesting improved empathy and reduced stigma.

**Table 4:** Medical Condition Regards Scale survey results (n (%)) OUD: opioid use disorder

Please answer the following with regard to patients with OUD	Strongly disagree	Disagree	Not sure, but probably disagree	Not sure, but probably agree	Agree	Strongly agree	Participants with improvement in regard from paired pre > post
I prefer not to work with patients like this.							
Pre	3 (13.0)	9 (39.1)	7 (30.4)	2 (8.7)	2 (8.7)	0 (0.0)	
Post	6 (28.6)	8 (38.1)	3 (14.3)	4 (19.1)	0 (0.0)	0 (0.0)	9 (50.0)
Patients like this irritate me.							
Pre	5 (21.7)	6 (26.1)	6 (26.1)	5 (21.7)	1 (4.4)	0 (0.0)	
Post	2 (9.5)	10 (47.6)	3 (14.3)	4 (19.1)	2 (9.5)	0 (0.0)	5 (27.8)
I enjoy giving extra time to patients like this.							
Pre	0 (0.0)	5 (21.7)	8 (34.8)	4 (17.4)	6 (26.1)	0 (0.0)	
Post	1 (4.8)	2 (9.5)	8 (38.1)	4 (19.1)	5 (23.8)	4 (19.1)	3 (33.3)
Patients like this are particularly difficult for me to work with.							
Pre	2 (8.7)	7 (30.4)	7 (30.4)	4 (17.4)	2 (8.7)	1 (4.4)	
Post	1 (4.8)	10 (47.6)	4 (19.1)	4 (19.1)	1 (4.8)	1 (4.8)	7 (38.9)
Working with patients like this is satisfying.							
Pre	1 (4.4)	4 (17.4)	8 (34.8)	4 (17.4)	6 (26.1)	0 (0.0)	
Post	0 (0.0)	3 (14.3)	6 (28.6)	5 (23.8)	6 (28.6)	1 (4.8)	8 (44.4)
I feel especially compassionate toward patients like this.							
Pre	0 (0.0)	2 (8.7)	7 (30.4)	7 (30.4)	7 (30.4)	0 (0.0)	
Post	0 (0.0)	0 (0.0)	7 (33.3)	7 (33.3)	4 (19.1)	3 (14.3)	9 (50.0)
I can usually find something that helps patients like this feel better.							
Pre	0 (0.0)	3 (13.0)	8 (34.8)	6 (26.1)	5 (21.7)	1 (4.4)	
Post	0 (0.0)	0 (0.0)	3 (14.3)	7 (33.3)	8 (38.1)	3 (14.3)	11 (61.1)
There is little I can do to help patients like this.							
Pre	1 (4.4)	14 (60.9)	4 (17.4)	3 (13.0)	1 (4.4)	0 (0.0)	
Post	4 (19.1)	13 (61.9)	3 (14.3)	1 (4.8)	0 (0.0)	0 (0.0)	7 (38.9)
Insurance plans should cover patients like this to the same degree that they cover patients with other conditions.							
Pre	0 (0.0)	1 (4.4)	1 (4.4)	2 (8.7)	12(52.2)	7 (30.4)	
Post	0 (0.0)	0 (0.0)	0 (0.0)	1 (4.8)	9 (42.9)	11 (52.4)	6 (33.3)
Treating patients like this is a waste of medical dollars.							
Pre	13 (56.5)	7 (30.4)	1 (4.4)	2 (8.7)	0 (0.0)	0 (0.0)	
Post	14 (66.7)	7 (33.3)	0 (0.0)	0 (0.0)	0 (0.0)	0 (0.0)	5 (27.8)

For example, the number of participants who disagreed/strongly disagreed with the statement "I prefer not to work with patients like this" increased from 52.1% pre-curriculum to 66.7% post-curriculum. Additionally, the tools provided in the curriculum for providers to help patients with OUD resulted in improvement in perceived ability to “find something that helps patients like this feel better,” with 51.2% of participants strongly agreeing/agreeing/probably agreeing with this statement in the pre-survey compared to 85.7% after the curriculum. 

Feedback received following the completion of the course was consistently positive. A majority of respondents (95.2%) felt the curriculum improved topic understanding, and 90.5% reported increased confidence in treating patients with OUD. The peer panel and lectures were rated as the most meaningful components of the curriculum. Qualitative feedback highlighted that the personal narratives shared by peers were particularly impactful. Participants discussed a better understanding of OUD and MOUD after the educational curriculum. Multiple participants described a desire to spend more time on shift with this patient population to “meet the patients where they are,” discuss harm reduction practices, prescribe MOUD, and connect them with peer support and resources.

## Discussion

The findings of this study highlight the efficacy and feasibility of a holistic OUD curriculum within a single conference day for EM residents. This educational intervention led to improvements in participants’ OUD-specific knowledge and attitudes toward individuals with OUD. Importantly, these gains were achieved within a single, structured didactic session, demonstrating the potential for integrating targeted, high-yield educational initiatives into existing residency education schedules without significant disruption.

A key outcome of the curriculum was the notable improvement in participants’ understanding of buprenorphine and comfort with prescribing MOUD, aligning with the curriculum’s objective. Notably, participants reported already feeling comfortable pre-curriculum in diagnosing OUD and recognizing withdrawal symptoms, suggesting that many EM clinicians may already possess foundational knowledge in identifying OUD but may lack comfort or training in its management. This finding underscores the need for more targeted training on management, including MOUD protocols.

The curriculum’s impact extended beyond reported knowledge acquisition as evidenced by the positive shift in participants’ attitudes toward individuals with OUD as measured by the MCRS. In particular, the peer panel emerged as a pivotal component of the curriculum, with qualitative feedback emphasizing the profound effect of personal narratives. This aligns with existing literature, which supports the use of storytelling and patient narratives as effective tools for exploring stigma and its effects [8]. Overall, these results suggest that this curriculum may help to foster a more compassionate, less stigmatizing approach toward individuals with OUD.

Limitations

This was a single-site, pilot study involving one EM residency program, and, therefore, participant numbers were relatively small, potentially limiting the generalizability to other settings. Additionally, the reliance on the self-reported nature of many of the knowledge and attitude assessments introduces potential for bias, particularly social desirability bias, which may influence survey responses, especially on the MCRS. Furthermore, while the study measured changes in knowledge and attitudes immediately after the curriculum, we did not assess long-term retention or objective changes in clinical behavior. Future studies should consider the incorporation of longer-term follow-up and objective measures of behavioral change, such as prescribing rates and patient outcomes.

## Conclusions

Amid the opioid epidemic and with recent changes removing prior barriers to prescribing MOUD, emergency physicians are uniquely positioned to intervene. This includes management of acute complications of OUD, initiation of MOUD, and connecting patients with other facilities that can continue to support recovery long-term. This OUD curricular intervention demonstrates the feasibility and value of a comprehensive OUD curriculum, resulting in improved participant knowledge and attitudes toward individuals with OUD. EM training should prioritize the integration and expansion of OUD education, given the ED's critical role as a point of care for individuals with OUD, often serving as their first - and sometimes only - interaction with the healthcare system.

## Appendices

Appendix A

**Table 5:** Sample peer panel interview questions

Panel questions
Briefly introduce yourself, including what your current role is and how you came to have that role.
What is a Peer and how would you define their/your role in an Emergency Department?
Can you share a personal experience of interacting with the healthcare system, specifically the emergency department? What were some positive aspects and what were some challenges you encountered?
What barriers have you faced when trying to access healthcare systems?
Can you describe any instances where you felt misunderstood or unheard by your healthcare provider? How did that impact your overall experience and willingness to seek care?
Were there any healthcare providers or systems that provided exceptional care or support during your journey? What made those experiences different/more positive?
Are there any resources or support networks you’ve found particularly helpful in navigating the healthcare system?
What suggestions do you have for Emergency Medicine physicians to better serve and support patients who have experienced similar challenges?
